# Peroxisomes and Disease - An Overview

**Published:** 2006-12

**Authors:** Hannah K. Delille, Nina A. Bonekamp, Michael Schrader

**Affiliations:** *Department of Cell Biology and Cell Pathology, University of Marburg, Robert Koch Str. 6, 35037 Marburg, Germany*

**Keywords:** carcinogenesis, fatty acid oxidation, genetic diseases, organelle biogenesis, peroxisome proliferation, reactive oxygen species

## Abstract

Peroxisomes are indispensable for human health and development. They represent ubiquitous subcellular organelles which compartmentalize enzymes responsible for several crucial metabolic processes such as β-oxidation of specific fatty acids, biosynthesis of ether phospholipids and metabolism of reactive oxygen species. Peroxisomes are highly flexible organelles that rapidly assemble, multiply and degrade in response to metabolic needs. Basic research on the biogenesis of peroxisomes and their metabolic functions have improved our knowledge about their crucial role in several inherited disorders and in other pathophysiological conditions. The goal of this review is to give a comprehensive overview of the role of peroxisomes in disease. Besides the genetic peroxisomal disorders in humans, the role of peroxisomes in carcinogenesis and in situations related to oxidative stress such as inflammation, ischemia-reperfusion, and diabetes will be addressed.

## INTRODUCTION ON PEROXISOMES

**Figure 1:**
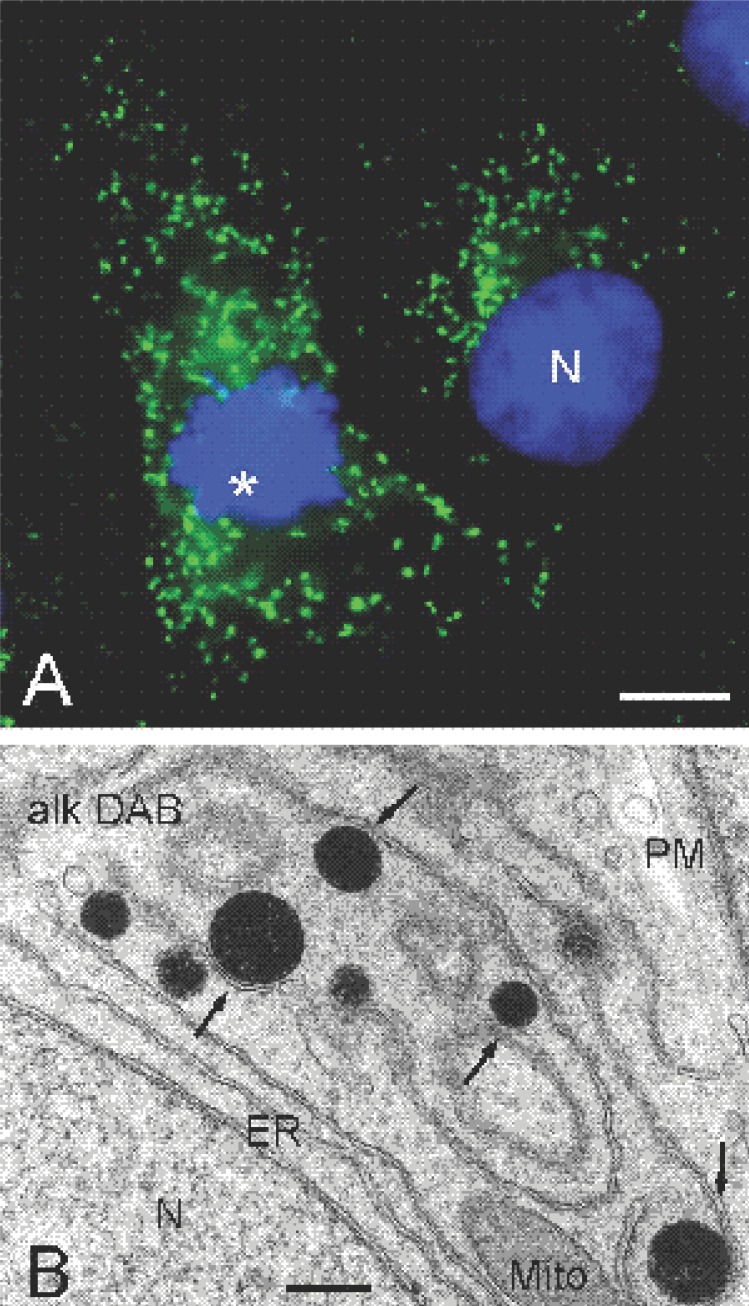
Appearance of mammalian peroxisomes in light- (A) and electron microscopy (B). (A) Fluorescence microscopy of peroxisomes in COS-7 cells. Peroxisomes (green) were stained with an antibody to PMP70, a peroxisomal membrane protein. Nuclei (blue) were stained with Hoechst 33258. Note the mitotic cell on the left (asterisk). (B) Ultrastructure of peroxisomes in rat hepatoma cells. Cytochemical localization of catalase was performed according to the alkaline DAB method (61). Note the close association of peroxisomes (black) with the smooth ER (arrows). N, nucleus; Mito, mitochondria; PM, plasma membrane; ER, endoplasmic reticulum. Bars, 10 μm (A), 500 nm (B).

Peroxisomes are crucial subcellular compartments for life of mammals including humans. They represent a class of ubiquitous and essential single-membrane bound cell organelles which play a critical role in a variety of metabolic processes, including fatty acid oxidation, ether phospholipid biosynthesis, peroxide and ROS metabolism, glyoxylate clearing, catabolism of purines, polyamines, prostaglandins and eicosanoids, and possibly the biosynthesis of isoprenoids (1, 2) (Fig. 1, Table 1). To fulfil these metabolic functions, mammalian peroxisomes harbour some 50 different enzyme activities. Several of these enzymes belong to the peroxisomal fatty acid oxidation system. Similar to mitochondria, mammalian peroxisomes possess their own fatty acid β-oxidation enzymes, which show substrate specificity, for example for very long chain fatty acids (VLCFA) or plant-derived branched chain fatty acids such as phytanic and pristanic acids, which can only be degraded in peroxisomes (3, 4). As the peroxisomal β-oxidation system is not able to degrade fatty acids to completion, the chain-shortened acyl-CoA esters have to be shuttled to mitochondria for full oxidation. Fatty acid oxidation in peroxisomes is a heat-generating process because peroxisomes lack a respiratory chain, and the peroxisomal FAD-linked oxidases donate their electrons directly to molecular oxygen to produce H_2_O_2_. Besides their important role in the oxidation of fatty acids, peroxisomes are required for the biosynthesis of important ether phospholipids, such as plasmalogens (5, 6). The first two steps of this pathway are exclusively peroxisomal, whereas the final steps are performed by enzymes of the ER. Although many peroxisomal enzymes and metabolic pathways have been well characterized, research on peroxisomal metabolism is still continuing. In addition to the metabolic enzymes, some 32 genes/proteins, so called peroxins (Pex), have been identified, which are required for the biogenesis and maintenance of functional peroxisomes in different species (7, 8).

**Table 1:** Metabolic functions of mammalian peroxisomes

Peroxide metabolism (catalase and H_2_O_2_-generating oxidases), ROS/NOS metabolism
Lipid biosynthesis (ether phospholipids/plasmalogens, bile acids, cholesterol and dolichol, fatty acid elongation)
Fatty acid β-oxidation (very long chain fatty acids, dicarboxylic acids, branched chain fatty acids, unsaturated fatty acids, arachidonic acid metabolism, and xenobiotic compounds)
Fatty acid α-oxidation (phytanic acid, xenobiotic compounds)
Catabolism of amino acids
Catabolism of polyamines
Catabolism of purines
Glyoxylate detoxification
Hexose monophosphate pathway

Peroxisomes are devoid of DNA or a protein translation machinery, and all of their proteins are encoded by nuclear genes. The majority of the peroxisomal proteins are synthesized on free polyribosomes in the cytoplasm and are post-translationally directed to the organelle (9). The sorting of peroxisomal matrix proteins is mediated by cytosolic receptors (Pex5p, Pex7p) that recognize two well-characterized classes of peroxisomal targeting signals (PTS1 and PTS2). The targeting and insertion of peroxisomal membrane proteins (PMPs) is less well understood (8, 10). It is suggested that Pex19p functions as a cycling receptor/chaperone for PMPs, which is recruited to the peroxisome by the membrane receptor Pex3p. Many of the identified peroxins are involved in the import of matrix proteins and contribute to the formation of the docking and translocation machinery at the peroxisomal membrane. Ongoing studies on peroxisome biogenesis/formation and protein import have so far revealed unique features of peroxisomes, which have often been in disagreement with existing dogmas in cell biology. Contrary to mitochondria and ER, for example, peroxisomal proteins can be imported in a completely folded or even oligomeric state, presumably via the formation of a transient membrane pore (11). Recent findings also indicate that peroxisomes can be formed de novo from the ER or a subdomain of the ER (12) in addition to growth and division of pre-existing peroxisomes (13, 14) (Fig. 1).

### Inherited disorders

The importance of peroxisomes for human health and normal development is underlined by the existence of several inherited diseases in humans, so called peroxisomal disorders (Table 2) (15-17). A defect in a peroxisomal gene can lead to a single enzyme deficiency which might affect one specific peroxisomal function or metabolic pathway. However, when the affected protein is a peroxin, which is involved in the biogenesis and maintenance of peroxisomes, several or all peroxisomal functions can be affected, and peroxisomes can be completely absent. This is the case in peroxisome biogenesis disorders (PBDs) (Table 2). As many peroxins are involved in protein import (targeting, docking, translocation, receptor recycling), a lack of matrix protein import is often observed, whereas the synthesis of peroxisomal membranes and import of membrane proteins appears to be normal. This results in the formation of “empty”, non-functional peroxisomal membranes, so called “ghosts”, which cannot fully develop and mature. The peroxisomal matrix proteins remain in the cytosol, where they cannot function or are degraded. Such a scenario has major consequences for most of the metabolic pathways located in peroxisomes. On one hand, an accumulation of peroxisomal substrates (e. g., VLCFA, plant-derived pristanic and phytanic acids, bile acid intermediates, pipecolic acid, an intermediary in lysine metabolism) occurs, which can only be handled by peroxisomes, and are toxic for the cell/organism. On the other hand, a shortage of end products of peroxisomal metabolism (e.g., ether glycerolipids/plasmalogens) is observed. Organs affected in most peroxisomal disorders include brain, spinal cord, or peripheral nerves, eye, ear, liver, kidney, adrenal cortex, Leydig cells in testis, skeletal system, and in some instances cardiovascular system, thymus, and pancreas (15).

**Table 2:** Inherited peroxisomal disorders

	Genes

Peroxisome biogenesis disorders	
Zellweger syndrome (ZS)	PEX1, PEX2, PEX3, PEX5, PEX6, PEX10, PEX13, PEX14, PEX16, PEX19, PEX26
Neonatal ALD (NALD)	PEX1, PEX5, PEX10, PEX26
Infantile Refsum’s disease (IRD)	PEX1, PEX2, PEX26
Rhizomelic chondrodysplasia punctata type 1 (RCDP type 1)	PEX7
Single protein defects	
X-linked adrenoleukodystrophy (X-ALD)	ABCD1
Contiguous ABCD1/DX1357E deletion syndrome	ABCD1, BCAP31
Pseudo-neonatal ALD (acyl-CoA oxidase deficiency)	ACOX
D-bifunctional protein deficiency/multifunctional protein 2 deficiency	HSD17B4
Acatalasaemia	CAT
Refsum’s disease (phytanol-CoA hydroxylase deficiency)	PAHX/PHYH
Rhizomelic chondrodysplasia punctata type 2 (DHAPAT deficiency)	GNPAT
Rhizomelic chondrodysplasia punctata type 3 (ADHAPS deficiency)	AGPS
Hyperoxaluria type 1 (Alanine glyoxylate aminotransferase deficiency)	AGXT
Mulibrey nanism	TRIM
α-Methylacyl-CoA racemase deficiency	AMACR
Glutaryl-CoA oxidase deficiency (glutaric aciduria type 3)	?

### Peroxisome Biogenesis Disorders (PBDs)

The fatal cerebro-hepato-renal syndrome of Zellweger (ZS), a developmental disorder with an incidence of 1:50,000 births, is the prototype and the most severe of the PBDs, characterized by the absence of functional peroxisomes (18). Other diseases belonging to the clinically and genetically heterogeneous PBD group are neonatal adrenoleukodystrophy (NALD), infantile Refsum’s disease (IRD), and rhizomelic chondrodysplasia punctata (RCDP) (2, 15, 16). ZS patients usually die within their first year of life, and suffer from neonatal hypotonia, craniofacial dysmorphy, hepatomegaly, renal cysts, adrenal atrophy, and profound neurological abnormalities, such as dys- or demyelination and neuronal migration defects. The body fluids of ZS patients contain high levels of bile acid intermediates, pipecolic and phytanic acid, and plasmalogen bisosynthesis as well as VLCFA β-oxidation are impaired. NALD, IRD and RCDP are also lethal disorders but present themselves more mildly resulting in a longer life span. Cell fusion studies using patient fibroblasts defined at least 13 complementation groups of PBDs caused by mutations in different PEX genes (19). Complementation group 1 is by far the largest, and is based on mutations in the Pex1 gene encoding an AAA-protein required for peroxisomal matrix protein import (Table 2).

### Peroxisomal single protein defects

The most common of the single enzyme defects, in which peroxisomes are present but a single enzyme function is deficient, is X-linked adrenoleukodystrophy (XALD). XALD (estimated incidence between 1:40,000 and 1:100,000) is based on mutations in the ALD gene encoding an ATP-binding cassette (ABC) transporter protein of the peroxisomal membrane, which is involved in the import/activation of VLCFA (20). Defects or a loss of ALD protein lead to an accumulation of VLCFA, and clinically to progressive demyelination/neurodegeneration in the central nervous system, adrenal insufficiency and death within a few years (21). Other hereditary deficiencies are based on mutations in individual β-oxidation enzymes or enzymes involved in ether phospholipid biosynthesis (Table 2) (2). Evidence derived from mouse models of peroxisomal β-oxidation (and PPARα, see below) deficiencies further highlight the importance of inducible peroxisomal fatty acid oxidation in energy metabolism, and in the development of steatosis and steatohepatitis (22). A deficiency of the peroxisomal enzyme alanine:glyoxylate aminotransferase (AGT), which catalyzes the transamination between L-alanine and glyoxylate thus forming pyruvate and glycine, leads to hyperoxaluria type 1 (23). This lethal disorder is characterized by an accumulation of glyoxylate and oxalate in tissues and body fluids, especially urine, leading to the precipitation of calcium oxalate and renal failure. In some patients the deficiency is based on an unusual mistargeting of AGT to mitochondria (24).

### Diagnosis and Therapy

Much progress in understanding the molecular defects and pathophysiology of the peroxisomal disorders has been made by studying peroxisome biogenesis in yeast mutants, by complementation analysis, and by the generation of knock-out mice. Great promise for the early diagnosis of PBDs lies in the molecular analysis of PEX genes, and molecular testing is evolving. Laboratory diagnosis usually involves blood and urine analysis (e. g., plasma VLCFA analysis, analysis of plasmalogens in erythrocytes, alpha-oxidation of phytanic acid) followed by detailed biochemical and morphological studies in patient's fibroblasts (15, 16). As abnormalities in PBD patients already develop in utero, the postnatal treatment options are limited. Therapies are mostly supportive, aiming to improve the developmental outcome, survival and quality of life. Strategies have been developed to correct the different biochemical abnormalities in patients with milder phenotypes and less pronounced abnormalities, for example by the reduction of accumulated precursors (dietary regimens to reduce VLCFA and phytanic acid) or replacing deficient products (supplementation of alkylglycerol, or docosahexaenoic acid) (25). In the case of XALD, pharmacological approaches (e.g., Lorenzo’s oil, immunosupression) or allogenic bone marrow transplantation have been performed (26). An alternative approach is the so called pharmacological gene therapy, which uses certain drugs (e. g., 4-phenylbutyrate) to increase the expression of peroxisomal genes which can either complement the function of the disease gene or increase the number and matrix protein content of peroxisomes (23, 27).

### Peroxisome proliferation and cancer

A remarkable and unique feature of peroxisomes is their ability to proliferate and multiply, or be degraded in response to nutritional and extracellular environmental stimuli. Peroxisome proliferation is usually characterized by an increase in the number and size of peroxisomes, and an induction of peroxisomal enzymes, especially those involved in fatty acid β-oxidation (3). The list of compounds inducing peroxisome proliferation, termed “peroxisome proliferators” (PPs), is quite long and includes hypolipidemic drugs, industrial chemicals such as plasticizers and lubricants, agrochemicals, toxic environmental pollutants as well as endogenous substances such as fatty acids (28). Selective transcription of peroxisomal genes by those compounds is mediated by the peroxisome proliferator activated receptor-α (PPARα), which belongs to the family of nuclear transcription factors (29), and acts as heterodimeric partner with retinoid X receptor at peroxisome proliferator response elements (PPREs). Some PPs are known as non-genotoxic carcinogens, which induce tumors (primarily in the liver) upon chronic administration to rodents (30). A proposed mechanism of liver tumor formation is the induction of sustained oxidative stress due to increased levels of H_2_O_2_ generation (via increased peroxisomal fatty acid β-oxidation) (31). Other mechanisms such as enhanced cell replication, promotion of spontaneous preneoplastic lesions, inhibition of apoptosis, and release of superoxide radicals from Kupffer cells have also been suggested (32-36). There is growing evidence that the activation of PPARα is involved in PP-induced liver growth and carcinogenesis in rodents (22, 36, 37). PPARα-/- mice are refractory not only to the peroxisome proliferating effect but are also resistant to hepatic carcinogenesis when fed a diet containing a potent non-genotoxic carcinogen such as WY-14,643 (38). Similarly, the low level of PPARα in livers of primates and humans (and other differences in the activity of PPARα) seems to be responsible for the resistance of those species to the carcinogenic effect of PPs (39), although their lipid lowering ability is not affected by that. However, the safety and cancer risk assessment of PPs to humans remains an issue of discussion (40).

### Peroxisomes, ROS and oxidative stress

It is now well established that peroxisomes have a key role in both the production and scavenging of reactive oxygen species (ROS) (41-43). Peroxisomes generate significant amounts of hydrogen peroxide (about 35% of all H_2_O_2_ produced in rat liver) through the action of several peroxisomal oxidases (e. g., their acyl-CoA oxidases), that can be converted to more aggressive ROS (44). However, peroxisomes also contain multiple antioxidant enzymes (e. g., catalase, Cu Zn-SOD, glutathione peroxidase, epoxide hydrolase, peroxiredoxin I, MnSOD) that contribute to the regulation of intracellular ROS levels and thus oxidative stress. The massive peroxisome proliferation induced by a variety of PPs and the subsequent tumor formation in rodents (see above) is evidently due to imbalance in the formation and scavenging of ROS generated by peroxisomes. Evidence for increased oxidative stress was also reported in fibroblasts of patients with multifunctional protein-2 deficiency (Table 2), a peroxisomal β-oxidation defect, but not in patients with a PBD (45). Furthermore, peroxisomes have been shown to undergo functional alterations (e. g., changes of peroxisomal volume density, altered activities of their antioxidant enzymes and peroxisomal β-oxidation) during various pathophysiological conditions that are associated with ROS production such as inflammation, ischemia-reperfusion, diabetes and hepatic allograft rejection thereby leading to an imbalance in the cellular redox state (46-49). The nuclear receptor PPARγ, which is a master regulator of adipogenesis as well as adipocyte metabolism, appears to be important in regulating cellular responses to oxidative stress, thus being at the crossroads of obesity, insulin resistance, and cardiovascular disease (50). Systemic fatty acid mobilisation in experimentally induced diabetes in rats is supposed to increase peroxisomal β-oxidation via PPARα, whereas catalase activity is reduced (51). Because of the central role of peroxisomes in the catabolism of inflammatory lipid mediators such as harmful metabolites of arachidonic acid or leukotriene B4 (52), the impairment of peroxisomal function can contribute significantly to the prolongation and intensification of an inflammatory process. In this respect the activation of PPARα by leukotriene B4 (53), which stimulates the oxidative degradation of fatty acids and their derivatives, serves to limit the inflammatory process and associated damaging effects (54). The stimulation of PPARα (and other PPARs) by different peroxisome proliferators/ligands can therefore also exert a protective function (50, 55, 56), thus underlining the importance of these pharmacological compounds in the development of novel therapeutic concepts (37, 57, 58).

## CONCLUSIONS

Peroxisomes are highly flexible organelles that rapidly assemble and degrade in response to metabolic needs. Their critical role in a variety of metabolic processes, especially in lipid metabolism, renders them essential for human health and development. Studies on the biogenesis of peroxisomes in yeast and mammalian model systems, on peroxisome proliferation, the generation of knock-out mice and complementation analysis has improved our understanding of the PEX genes and molecular mechanisms responsible for a wide spectrum of clinically and genetically diverse inherited peroxisomal disorders. These ongoing studies will promote new approaches for diagnosis and therapy. The improved understanding of the PPAR-mediated mechanism of peroxisome proliferation, the central role of PPARα in the carcinogenic activity of peroxisome proliferators, and the role of PPARα as an important regulator of inflammation has stimulated the investigation of the risks and benefits of peroxisome proliferators as therapeutical agents. Such investigations further highlight the crucial role of peroxisomes in lipid and ROS metabolism and homeostasis, and link functional alterations of peroxisomes to pathophysiological conditions like inflammation, ischemia-reperfusion, carcinogenesis and diabetes. Other aspects of peroxisome biogenesis, such as division (13, 14) and movement (59) have so far not been related to diseases. An impairment of the peroxisomal fission machinery, a loss of trafficking and disturbed cytoplasmic distribution of peroxisomes might lead to a regional loss of essential peroxisomal activities and thus, to cell damage and degeneration. In line of this, overexpression of the microtubule-associated protein tau, which inhibits kinesin-dependent transport of peroxisomes (and of other organelles, neurofilaments, and vesicles), increases the susceptability of neurons to oxidative stress (60). Despite all the advances made in the past several years, many details of peroxisomal functions remain to be resolved, and a whole range of old and new questions have to be answered, thus keeping these fascinating organelles in the limelight.

## Abbreviations

ALD: adrenoleukodystrophy
ER: endoplasmic reticulum
PBD: peroxisome biogenesis disorder
PEX: peroxin
PMP: peroxisomal membrane protein
PP: peroxisome proliferator
PPAR: peroxisome proliferator activated receptor
PTS: peroxisomal targeting signal
ROS: reactive oxygen species
